# Invasive Potential of Melanoma Cells Correlates with the Expression of MT1-MMP and Regulated by Modulating Its Association with Motility Receptors via N-Glycosylation on the Receptors

**DOI:** 10.1155/2014/804680

**Published:** 2014-08-11

**Authors:** Amit Ranjan, Rajiv D. Kalraiya

**Affiliations:** Advanced Centre for Treatment, Research and Education in Cancer (ACTREC), Tata Memorial Centre, Sector 22, Kharghar, Navi Mumbai 410210, India

## Abstract

Matrix remodeling and invasion of basement membrane are the major determinants of malignant progression. Matrix degrading enzymes play a pivotal role in this process and have been shown to be regulated at multiple levels. Using high metastatic B16F10 and its invasive variant B16BL6 cells, we previously demonstrated that the expression of *β*1,6 branched N-oligosaccharides promotes cellular adhesion on different matrix components which in turn induces secretion of MMP9. The present investigations report that although the two cell lines do not differ in the expression of uPAR, expression of MT1-MMP is significantly higher on B16BL6 cells. Analysis of the transcripts of tissue inhibitors of matrix metalloproteinases (TIMPs) showed that expression of both TIMP1 and TIMP2 correlates negatively with the invasive potential of cells. CD44 and *β*1 integrin, the two important receptors involved in motility, were identified to carry *β*1,6 branched N-oligosaccharides in an invasive potential dependent manner. However, their glycosylation status did not appear to influence their surface expression. Although glycosylation on CD44 had no effect, that on *β*1 integrin significantly affected association of *β*1 integrin with MT1-MMP. The results thus demonstrate that the cancer cells use multiple mechanisms for degradation of matrix in a controlled manner to couple it with movement for effective invasion.

## 1. Introduction

Invasion is a key requirement for malignant progression and remodeling of extracellular matrix is a major component of the invasion process [1]. However, invasion is a complex process that requires modulation of cellular adhesiveness to the substratum. Adhesion influences not only movement but also signaling that determines the secretion of different matrix degrading enzymes. Most of the enzymes are secreted in the zymogenic forms that need to be activated in the vicinity of the invading cancer cells which in turn help in preventing indiscriminate degradation of matrix. The degraded matrix components aid directional motility by serving as chemoattractants for invading cancer cells [2].

Matrix remodeling is majorly accomplished by a group of enzymes like proteoglycanases and proteases, responsible for degrading different components of the matrix. Cathepsins, serine proteases, and metalloproteinases are the major classes of proteases involved in cancer cell invasion [3]. Although these enzymes are very critical for invasion and metastasis of cells, their expression, activation, and functional properties are tightly regulated. The levels of expression of these enzymes, thus, often do not correlate with the invasiveness of the cells or with disease progression. For example, regulation of matrix degradation by matrix metalloproteinases (MMPs) can occur at several levels such as at the level of (i) basal secretion of MMPs, (ii) induction of their secretion in response to external cues, namely, adhesion to extracellular matrix (ECM) or basement membrane (BM) components, (iii) localization towards invasive front and their activation in the proximity of the invading cell, or (iv) inhibition of their activity by specific metalloproteinase inhibitors like TIMPs [4]. Expression of the metalloproteinases positively correlates with metastatic and invasive potential of several cancers such as breast and colorectal cancers [5, 6]. However, it is not always the case. For instance, expression of gelatinases, a class of matrix metalloproteinases, did not correlate with the metastatic/invasive potential of B16 melanoma variants [7, 8].

Previously, we showed that although the basal secretion of MMP2 and MMP9 remains almost similar between the invasive variants [8], increased adhesion of highly invasive cells on different matrix components induces increased secretion of MMP9. Increased expression of *β*1,6 branched N-oligosaccharides on integrin receptors like *β*1 appears to promote adhesion and thus MMP9 expression [9]. The present studies aim to investigate the expression and localization of MT1-MMP and uPAR with receptors carrying *β*1,6 branched N-oligosaccharides and the role of these oligosaccharides in regulating their association with receptors involved in motility. Most of the MMPs are secreted in the zymogenic form and cancer cells utilize different mechanisms to activate them in the vicinity of the cancer cells. One of the mechanisms is by colocalizing the molecules involved in activating the proenzymes with the receptors involved in movement. Plasmin is one of the major enzymes responsible for converting the proMMPs into their active form. The plasmin is generated from plasminogen in the vicinity of the cancer cells by the action of urokinase plasminogen activator (uPA) which is often localized in the invading front via its receptor (uPAR) [10]. Membrane tethered forms of MMPs like MT1-MMP are another class of proteins present on the membrane in the active form (due to the action of furin), that convert proMMPs into active forms [11, 12].

By comparing the parent B16F10 and its invasive variant B16BL6, the present investigations demonstrate that CD44 and *β*1 integrin carry *β*1,6 branched N-oligosaccharides. However, their overall expression or that on the cell surface was independent of their glycosylation status or the invasiveness of the cell lines. Although there was no difference in the levels of uPAR expression, the invasive variant showed significantly increased surface and overall expression of MT1-MMP. Further MT1-MMP was shown to colocalize with the *β*1 subunit of integrin receptors in a glycosylation dependent manner. This most likely promotes invasion by converting excess MMP9, secreted in response to increased adhesion, into active form in the close proximity of the cancer cells, most likely in the invading front.

## 2. Materials and Method

### 2.1. Cell Lines and Reagents

B16F10 and B16BL6 murine melanoma cell lines were a kind gift from Professor I. J. Fidler, MD Anderson Cancer Centre, Houston, USA. Cell culture reagents were from Invitrogen, USA. PVDF membrane and the ECL kit were purchased from GE Healthcare, Amersham, UK. Culture ware was from BD Falcon, USA. Rat anti-mouse *β*1 integrin monoclonal antibody (clone Mab1.2) and mouse anti-mouse MT1-MMP monoclonal antibody (clone 113-5B7) were obtained from Calbiochem, USA. Rat anti-CD44 (IM-7) monoclonal antibody was a kind gift from Dr. Jayne Lesley. Goat anti-mouse uPAR antibody was obtained from R and D Systems, USA. Biotinylated lectin leucophytohemagglutinin (L-PHA), L-PHA agarose beads, and Vectashield Mounting Medium were obtained from Vector Labs, USA. All other chemicals were of analytical grade and were purchased locally.

### 2.2. Cell Culture

Melanoma cells were routinely cultured as described in [9].

### 2.3. Preparation of Total Cell Lysates and Western Blotting

Total cell lysates were prepared exactly as described in [13] using lysis buffer containing 10 mM Tris chloride, 150 mM NaCl, 1% NP-40, 0.5% deoxycholate, 1 mM each of MgCl_2_ and CaCl_2_, and protease inhibitors (1 *μ*g/mL each of pepstatin, leupeptin, and aprotinin and 0.3 mM PMSF). The protein content was estimated as per [14] and was separated on SDS-PAGE according to [15] and transferred onto PVDF membrane as described in [16].

### 2.4. Purification of L-PHA Reactive Proteins

L-PHA reactive proteins are purified as described in [17].

### 2.5. Flow Cytometric Analysis

For flow cytometry, 90% confluent melanoma cells were harvested as described above. Cells were washed thrice with phosphate buffered saline (PBS) and then fixed with 1% paraformaldehyde in PBS (pH 7.4) by overnight incubation at 4°C. The cells were pelleted and washed 3x with PBS to remove fixative followed by blocking with FACS buffer (1% FBS in PBS). Subsequently, surface expression of membrane proteins such as CD44 and *β*1 integrin was analyzed by incubating with their specific primary antibodies for 1 h. This was followed by incubation with fluorophore tagged secondary antibody, respectively. Labeled cells were analyzed using FACS caliber (BD Biosciences) and data was analyzed using CellQuest Pro software.

### 2.6. Colocalization Using Laser Confocal Microscopy

B16BL6 cells were seeded on coverslips and grown overnight in complete medium up to 70–80% confluence. Cells were washed thrice with PBS (pH 7.4) and fixed with 2% paraformaldehyde at RT for 5 min. Cells were washed again with PBS and blocked with 3% BSA in PBS for 1 h at RT in humidified chamber. Cells were incubated with primary antibodies for 1 h in humidified chamber, followed by three washes with PBS to remove excess or nonspecifically bound antibody. Cells were further incubated with fluorescent tagged secondary antibodies for 1 h followed by three washes with PBS. Cells incubated only with fluorescent tagged secondary antibody serve as isotype-control. Nuclei were stained with 5 *μ*g/mL of DAPI in PBS for 1-2 min and coverslips were mounted on slides using Vectashield Mounting Medium (Vector Labs). Images were acquired using a Carl Zeiss laser confocal microscope at 63x magnification. Images were analyzed using ImageJ 1.43 software (NIH).

### 2.7. Reverse Transcription and Semiquantitative-PCR

Total RNA was isolated from melanoma cell lines using Trizol reagent according to the manufacturer's instructions. RNA concentration was determined by measuring absorbance at 260 nm using NanoDrop 1000 spectrophotometer. The first strand cDNA was synthesized by ProtoScript First cDNA Synthesis Kit using oligo(dT) primers and M-MuLV reverse transcriptase as per manufacturer's protocol. Following forward and reverse primer sequences primers were used to check the expression of uPAR, MT1-MMP, TIMP1 and TIMP2, and GAPDH. GAPDH served as loading control. uPAR: 5′-CACAAACCTCTGCAACAGGC-3′ and 5′-GTAGCCACCAGGCACTGATT-3′, respectively. MT1-MMP: 5′-AGTAAAGCAGTCGCTTGGGT-3′ and 5′-TGGGTAGCGATGAAGTCTTC-3′, respectively. TIMP1: 5′-GGCATCTGGCATCCTCTTGT-3′ and 5′-ACTCTTCACTGCGGTTCTGG-3′. TIMP2: 5′-GAGATCAAGCAGATAAAGATG-3′ and 5′-GACCCAGTCCATCCAGAGGC-3′, respectively.

GAPDH: 5′-TGAAGGTCGGTGTGAACGGATTTG-3′ and 5′-CATGTAGGCCATGAGGTCCACCAC-3′, respectively.

## 3. Results

### 3.1. Expression of Membrane Tethered Matrix Metalloproteinase-1 (MT1-MMP) Correlates with Invasive Potential While Urokinase Plasminogen Activation Receptor (uPAR) Expression Remains Unaltered

**Figure 1:**
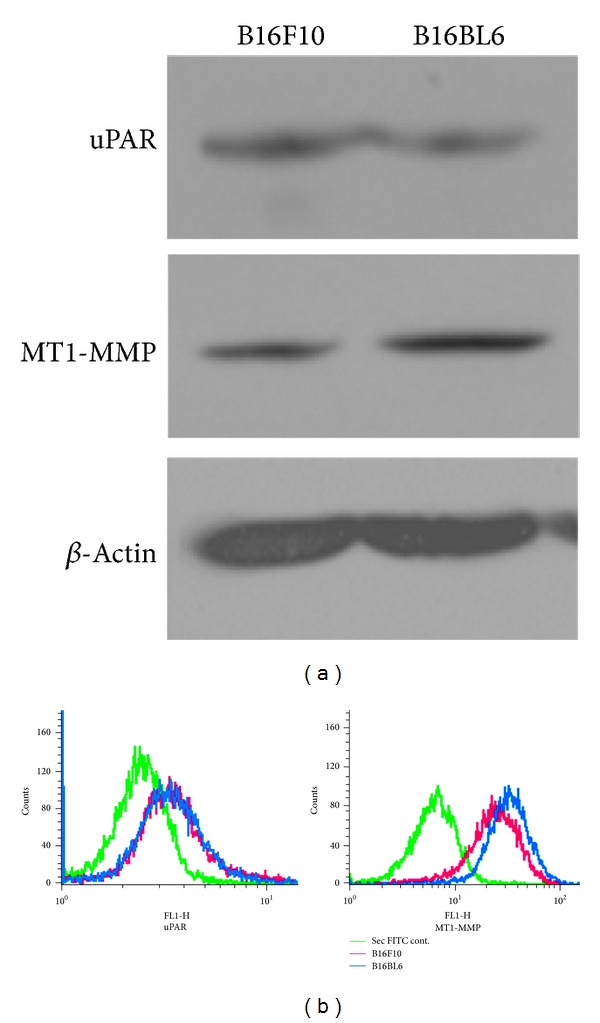
Analysis of the expression of membrane tethered protease or receptor of protease. (a) Cell lysates from B16F10 and B16BL6 were blotted and probed with antibodies for MT1-MMP and uPAR. *β*-Actin served as loading control. (b) Flow cytometric analysis of the surface expression of MT1-MMP and uPAR in B16F10 and B16BL6 cells. Cells treated with only secondary FITC served as control.

MT1-MMP and uPAR are the major molecules which are involved in regulating the activity of the MMPs. Their expression has also been shown to be altered in invasive and metastatic cancer cells. Cell lysates from B16F10 and B16BL6 cells were blotted and probed with MT1-MMP and uPAR specific antibody. Results showed that expression of MT1-MMP correlates positively with the invasiveness. However, the expression of uPAR did not correlate with invasiveness (Figure 1(a)). This was further demonstrated by analyzing the surface expression of MT1-MMP and uPAR by flow cytometry which showed similar results (Figure 1(b)).

### 3.2. Analysis of the Transcripts Confirms That the Levels of MT1-MMP Correlate Positively While Those of TIMPs Correlate Negatively with Invasive Potential of Melanoma Cells

**Figure 2:**
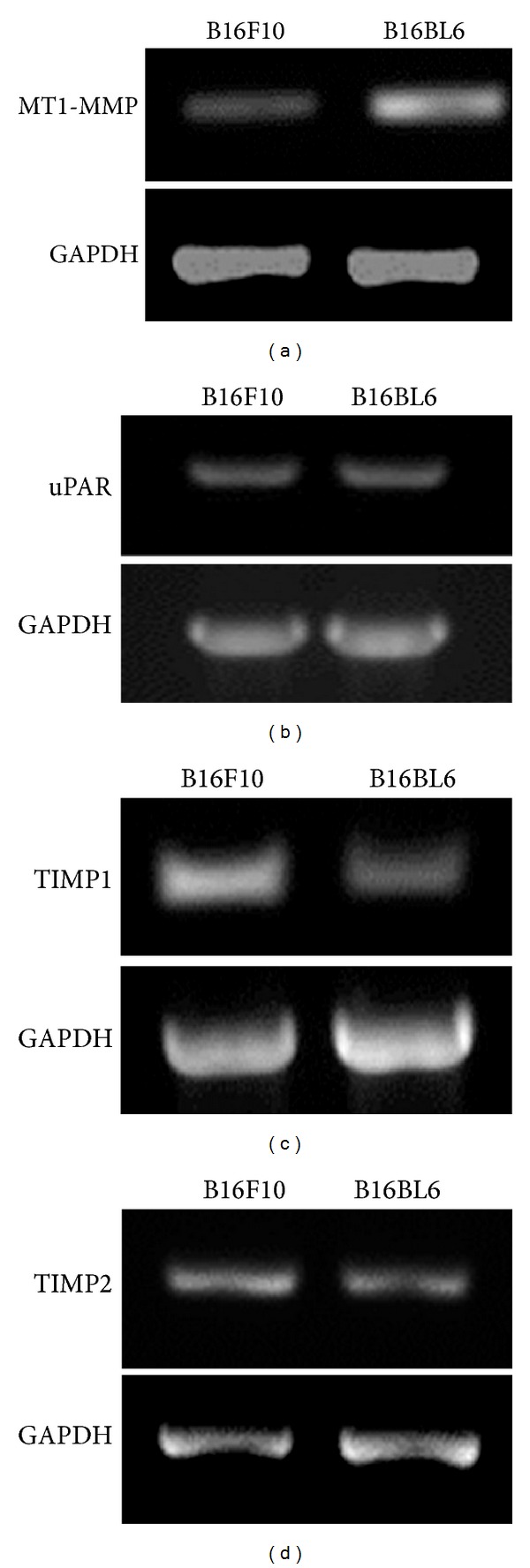
Analysis of the transcript levels of uPAR, MT1-MMP, TIMP1, and TIMP2. Transcript levels of (a) MT1-MMP, (b) uPAR, (c) TIMP1, and (d) TIMP2 were analyzed in B16F10 and B16BL6 cells by semiquantitative RT PCR. GAPDH served as loading control.

To confirm that the increased expression of MT1-MMP correlates with invasive properties of B16BL6 cells, we checked the expression of MT1-MMP and uPAR at transcript level in B16F10 and B16BL6 cells. Results showed that expression of MT1-MMP transcript is more in B16BL6 cells as compared to B16F10 cells (Figure 2(a)). However, transcript level of uPAR is unaltered in these two cell lines (Figure 2(b)). Analysis of endogenous inhibitor of MMPs, TIMP1, and TIMP2 in these cell lines showed that the levels of their transcripts are significantly lower in B16BL6 cells as compared to B16F10 cells (Figures 2(c) and 2(d)). Another mechanism by which tumor cells regulate matrix degradation is by regulating the association of uPAR and MT1-MMP with motility receptors.

### 3.3. Motility Receptors CD44 and *β*1 Integrin Carry *β*1,6 Branched N-Linked Oligosaccharides; However Their Surface Expression Is Independent of Their Glycosylation Status

**Figure 3:**
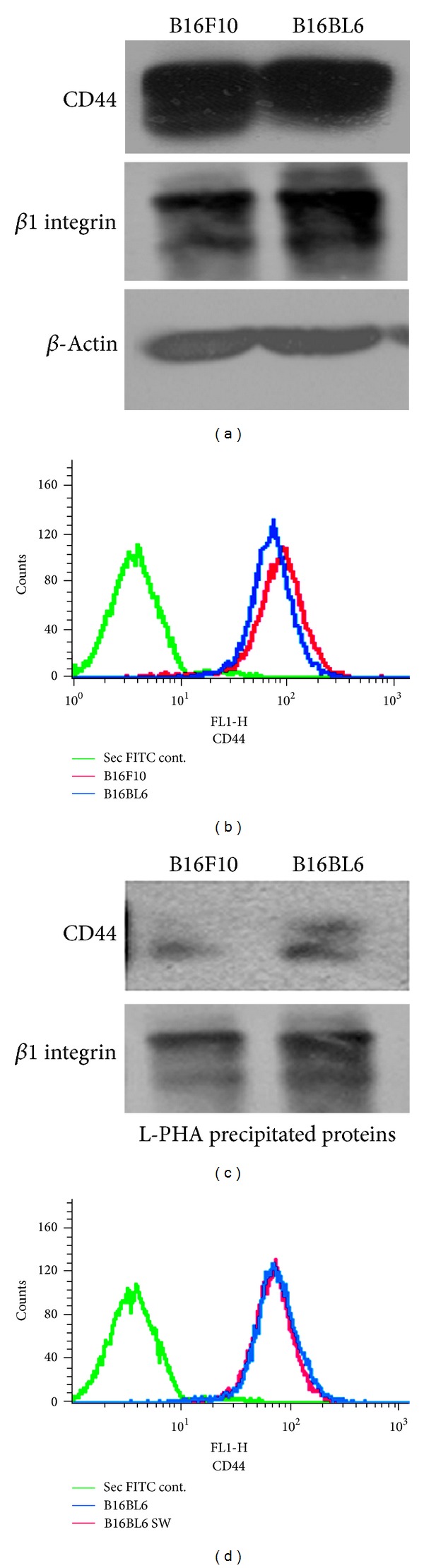
Motility receptors CD44 and *β*1 integrin carry *β*1,6 branched N-oligosaccharides and inhibition of glycosylation does not influence their surface expression. (a) Cell lysate from B16F10 and B16BL6 cells was blotted and probed with CD44 and *β*1 integrin specific antibodies. *β*-Actin served as equal loading control (common for both Figures 1(a) and 3(a)). (b) Flow cytometric analysis of the surface expression of CD44 from B16F10 and B16BL6 cells. (c) Lectin L-PHA precipitated proteins from the cell lysates of B16F10 and B16BL6 cells were blotted and probed with *β*1 integrin and CD44 specific antibodies. (d) Flow cytometric analysis of the surface expression of CD44 from B16BL6 and the same cells treated with SW (B16BL6 SW). Cells treated with only secondary FITC served as control.

CD44 and integrin receptors with *β*1 subunit are the major motility receptors that regulate matrix degradation by associating with molecules/receptors involved in matrix degradation [18]. The expression of both CD44 and *β*1 integrin did not correlate with invasive potential of the cells as both B16F10 and B16BL6 cells express them in almost equal amounts (Figure 3(a)). Their expression on the surface of these two cell lines is also identical for CD44 as shown in Figure 3(b) and for *β*1 integrin as shown previously [9]. Lectin L-PHA precipitation and western blotting of proteins from cell lysates of both B16F10 and B16BL6 cells showed that both *β*1 integrin and CD44 carry *β*1,6 branched N-oligosaccharides. Proteins from B16BL6 cells showed higher levels of precipitation of both CD44 and *β*1 integrin with L-PHA suggesting that these proteins carry higher levels of *β*1,6 branched N-oligosaccharides. However, inhibition of glycosylation with swainsonine (SW) did not have any effect on the surface expression of CD44 (Figures 3(c) and 3(d)) and even *β*1 integrin [9]. However, role of *β*1,6 branched N-oligosaccharides on the motility receptors in regulating their association with MT1-MMP needs to be assessed.

### 3.4. Role of *β*1,6 Branched N-Linked Oligosaccharides in Regulating the Localization of Membrane Tethered MT1-MMP and uPAR with Motility Receptors *β*1 Integrin and CD44

**Figure 4:**
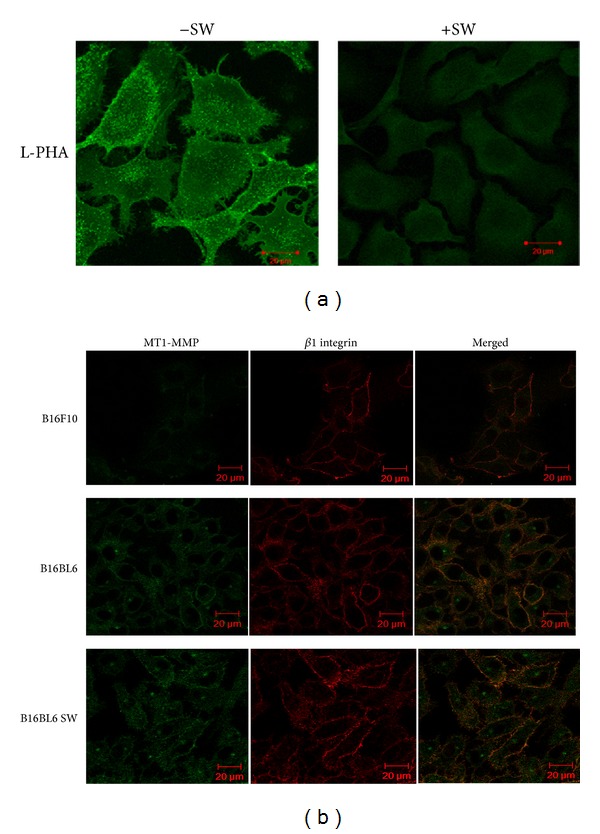
Effect of glycosylation on regulating association of *β*1 integrin and MT1-MMP. (a) Biotinylated lectin L-PHA staining of B16BL6 (−SW) and the same cells treated with swainsonine (+SW). (b) Colocalization of MT1-MMP (green) with *β*1 integrin (red) in B16F10, B16BL6, and B16BL6 cells treated with SW (B16BL6 SW). Colocalized or merged images were shown in yellow. Scale bar 20 *μ*m.

Focalized degradation of ECM and BM is very crucial for invasion and it is highly regulated at various levels. To achieve this, tumor cells localize the proteases with motility receptors towards the invading front. Therefore, role of glycosylation in regulating protease localization towards invading front was investigated. *β*1,6 branched N-linked oligosaccharides were inhibited using SW as seen by biotinylated L-PHA staining (Figure 4(a)). Results showed decreased association of MT1-MMP with that of *β*1 integrin in B16F10 as compared to B16BL6 cells (Figure 4(b), upper panel) which was also evident from the low expression of MT1-MMP in B16F10 cells. Upon inhibition of glycosylation by SW in B16BL6 cells, the association of *β*1 integrin with MT1-MMP was decreased as studied by confocal microscopy (Figure 4(b)).

**Figure 5:**
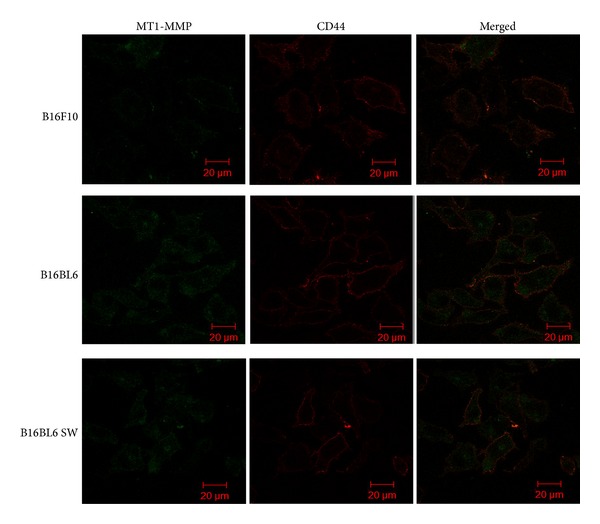
Effect of glycosylation on regulating association of CD44 and MT1-MMP. Colocalization of MT1-MMP (green) with CD44 (red) in B16F10, B16BL6, and B16BL6 SW cells. Colocalized or merged images were shown in yellow. Scale bar 20 *μ*m.

**Figure 6:**
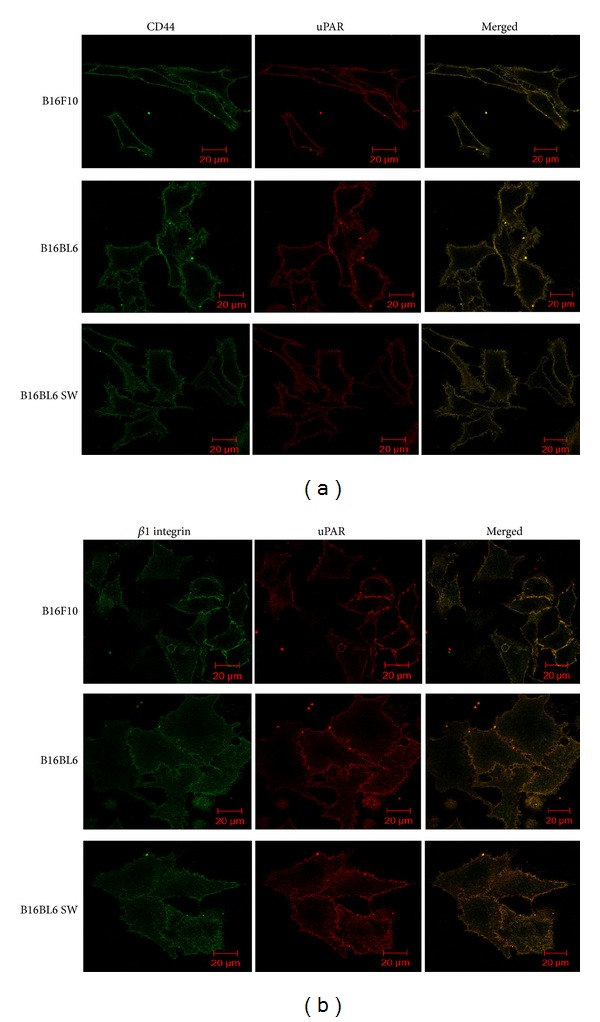
Effect of glycosylation on regulating association of motility receptors and uPAR. (a) Colocalization of CD44 (green) with uPAR (red) in B16F10, B16BL6, and B16BL6 SW cells. (b) Colocalization of *β*1 integrin (green) with uPAR (red) in B16F10, B16BL6, and B16BL6 SW cells. Colocalized or merged images were shown in yellow. Scale bar 20 *μ*m.

Moreover, association of *β*1 integrin with uPAR and CD44 with either MT1-MMP or uPAR was unaffected in untreated B16BL6 and those treated with swainsonine (B16BL6 SW) (Figures 5 and 6). Similarly, less association of MT1-MMP with CD44 in B16F10 cells as compared to B16BL6 cells was due to reduced expression of MT1-MMP in B16F10 cells (Figure 5, upper panel). However, these results would need to be confirmed by coimmunoprecipitation which was not possible because of nonavailability of good quality antibodies for immunoprecipitation.

## 4. Discussion

Invasion is the key determinant of cancer cell metastasis. It is required at all the different stages of metastatic cascade, namely, to breach the organ basement membrane, intravasation, and extravasation and for colonizing the secondary organ site [19]. Matrix degrading enzymes play a key role in invasion process and MMPs are the major participants in the process. (i) Owing to vast range of substrates they can act on, (ii) MMPs have large repertoire of soluble as well as membrane tethered forms [1]. However, their activity is regulated in diverse ways. Previously we showed that although the level of their expression does not correlate with invasive potential of melanoma variants, increased adhesion of highly invasive B16BL6 cells induced increased secretion of MMP9 [8, 9].

Most of the MMPs are secreted in the zymogenic form and therefore it needs to get activated. MT1-MMP and uPAR are the major molecules involved in the activation of MMPs and their expression has been shown to correlate with metastatic and invasive potential of several cancers [20–22]. MT1-MMP plays an important role in the activation of MMPs as they already get activated via furin during their transport to the cell surface [12]. Trimolecular complex of MT1-MMP, TIMP2, and proMMP2 has been shown to play an important role in activation of proMMP2 by the adjacent TIMP2-free MT1-MMP. Increased activation of MMP2 in turn activates proMMP9 [11]. Inhibition of the expression of MT1-MMP by its downregulation or by inhibiting its vesicular trafficking to cell surface has been shown to retard matrix degradation [23, 24]. Present investigations show that the total expression of MT1-MMP and on the cell surface indeed correlate with the invasive potentialof melanoma cells (Figures 1(a), 1(b), and 2(a)). Plasmin is the other major enzyme that is responsible for activation of MMPs. Urokinase plasminogen activator (uPA) gets localized to the cell surface via its receptor uPAR and controls the conversion of plasminogen into plasmin in close proximity to the cell surface to facilitate focalized activation of MMPs via plasmin [25]. Inhibition of uPAR and MMP9 expression has been shown to inhibit invasion in a glioblastoma cell line [26]. However, the levels of uPAR in these melanoma variants remained unaltered (Figures 1(a), 1(b), and 2(b)).

The activity of activated MMPs can also be regulated by the expression of tissue inhibitor of matrix metalloproteinases (TIMPs). Expression of TIMPs has been shown to influence the metastatic and invasive properties of cancer cells [27]. Analysis of the transcripts of both TIMP1 and TIMP2 was found to correlate negatively with invasiveness whereas that of MT1-MMP correlated with their invasive potential (Figures 2(a), 2(c), and 2(d)). Moreover, overexpression of TIMP1 in B16F10 cells has been shown to inhibit their ability to form metastatic colonies in lungs [28, 29]. This suggests that the increased secretion of MMP9 in response to increased adhesion to matrix is activated by increased surface MT1-MMP near the cell surface and MMP9 remains active in the absence of TIMPs.

For effective invasion, the matrix degradation is very often restricted towards the invading front by restricting the localization of MT1-MMP and uPAR with the motile machinery [24, 30, 31]. Integrins and CD44 are the major receptors that together bind to major components of the matrix and *β*1 integrin is an important component of most of the integrin receptors that bind to ECM and BM components [32]. Expression of motility receptors often gets altered as the tumor cells become metastatic and invasive. However, in B16 melanoma invasive variants, expression of motility receptors like *β*1 integrin [9] and CD44 remains unaltered (Figures 3(a) and 3(b)).

Both CD44 and *β*1 integrin have been shown to be among the major carriers of *β*1,6 branched N-oligosaccharides (Figure 3(c)). Expression of these oligosaccharides has been shown to be associated with invasive normal as well as cancer cells. Highly invasive cancer cells express such oligosaccharides on their invasive front [33–38]. We earlier showed that these oligosaccharides regulate adhesion, chemotaxis, and haptotactic motility of melanoma cells in a complex manner [8, 9, 17]. Recently, it has been shown that these oligosaccharides regulate association of uPAR with *α*v*β*3 in human melanoma cell lines [39]. To investigate if *β*1,6 branched N-oligosaccharides on CD44 and *β*1 integrin regulate matrix degradation by modulating their association with MT1-MMP and uPAR, the invasive B16BL6 cells were compared with the parent B16F10 cells or with B16BL6 cells treated with glycosylation inhibitor swainsonine (SW). Glycosylation on CD44 had no impact on its association with either MT1-MMP or uPAR (Figures 5 and 6(a)) while that on *β*1 integrin significantly promoted its association with MT1-MMP and was significantly inhibited on SW treatment (Figure 4(b)). The glycosylation however did not influence the association of *β*1 integrin with uPAR (Figure 6(b)).

These studies thus highlight that increased overall and surface expression of MT1-MMP and regulation of its association with integrin receptors with *β*1 subunits by *β*1,6 branched N-oligosaccharides may play a key role in generating activated MMP9 in the vicinity of migrating cells. Decreased expression of TIMPs may contribute towards maintaining them in activated forms.

## 5. Conclusions

These studies provide a clear evidence for the mechanisms used by melanoma cells to achieve an invasive phenotype. It demonstrates that the increased MMP9 secreted as a result of increased adhesion may be activated by MT1-MMP overexpressed on the cell surface. The focalized matrix degradation is ensured by downregulating TIMPs and regulating the association of MT1-MMP with motility receptor *β*1 integrin via *β*1,6 branched N-oligosaccharides.
